# Prediction of pathogenic mutations in human transmembrane proteins and their associated diseases via utilizing pre-trained Bio-LLMs

**DOI:** 10.1038/s42003-025-08452-7

**Published:** 2025-07-15

**Authors:** Lexin Cao, Lijun Quan, Qiufeng Chen, Bei Zhang, Zhijun Zhang, Liangchen Peng, Junkai Wang, Yelu Jiang, Liangpeng Nie, Geng Li, Tingfang Wu, Qiang Lyu

**Affiliations:** 1 School of Computer Science and Technology, Soochow University, Suzhou, China; 2 Collaborative Innovation Center of Novel Software Technology and Industrialization, Nanjing, China; 3 Jiangsu Key Laboratory of Drug Discovery and Translational Research for Brain Diseases, Soochow University, Suzhou, China

**Keywords:** Computational biology and bioinformatics, Protein function predictions, Machine learning

## Abstract

Missense mutations can disrupt the structure and function of membrane proteins, potentially impairing key biological processes and leading to various human diseases. However, existing computational methods primarily focus on binary pathogenicity classification for general proteins, with limited approaches specifically designed for membrane proteins, and even fewer methods capable of fine-grained, multi-label classification for specific disease categories. To address this gap, we proposed MutDPAL, a deep learning method specifically designed to identify pathogenic mutations in membrane proteins and further classify such pathogenic mutations into potential diseases categories. MutDPAL utilizes two pre-trained biological large language models (Bio-LLMs), one for raw sequence features and the other for encoding transmembrane environment features. By employing a cross-attention-based disease-protein association learning approach in the context of membrane proteins, MutDPAL captures the intricate relationships between mutations and diseases, enabling accurate pathogenicity prediction and classification into 15 distinct disease categories. Experimental results demonstrate that MutDPAL outperforms existing methods in predicting membrane protein mutation pathogenicity and excels in multi-label disease classification tasks, achieving high predictive accuracy across all 15 disease categories. MutDPAL is the first to combine transmembrane environment with disease encoding features for fine-grained disease classification, offering valuable insights into the pathogenicity of missense mutations in membrane protein.

## Abstract (web summary)

MutDPAL leverages pre-trained biological large language models to integrate transmembrane environment with disease encoding features, enabling fine-grained classification of pathogenic mutations in transmembrane proteins across 15 disease categories.

## Introduction

Membrane proteins constitute approximately one-quarter of the human proteome and play essential roles in maintaining cellular structure, signal transduction, and substance transport^1^. In fact, approximately 50% to 60% of membrane proteins are used as drug targets and are the basis for the treatment of many diseases^2^. Due to their unique structural properties, membrane proteins are particularly susceptible to genetic variations^3^. Single nucleotide variants, the most common type of variation in the human genome, can result in single amino acid substitutions (SAVs) when they occur in protein-coding regions. These missense mutations can alter protein structure and function, potentially causing human diseases^4,5^, some specific missense mutations in membrane proteins lead to nervous system diseases, immune system diseases (ISD), cancer and many other serious diseases^3,6^. Due to the limitations of existing studies, the known pathogenic missense mutations may represent only a fraction of the actual number of such mutations^7^. Furthermore, experimental characterizing these mutations require significant financial and material resources. Therefore, developing innovative computational methods to quickly and accurately predict the pathogenic effects of missense mutations is critical.

Over the past few years, numerous computational methods have been developed to predict the pathogenic effects of missense mutations. With advances in sequencing technology and the accumulation of experimental data, machine learning (ML) has become a dominant approach in pathogenicity prediction^8,9^. These methods generally fall into two categories: shallow ML and deep learning (DL). Early research primarily focused on shallow ML techniques, leveraging classical algorithms such as Support Vector Machines^10,11^, Random Forests (RF)^12–15^, and Gradient Boosting e.g., ref. ^16^, which rely on manually designed features, such as sequence, structural, or functional properties, and employed trained classifiers to make predictions. With the rise of DL, research has gradually shifted towards utilizing more complex models and data-driven feature learning methods, which have demonstrated considerable advantages in pathogenicity prediction tasks. For example, Varipred uses pre-trained protein language model (PLM) to extract sequence representations for predicting the pathogenicity of missense mutations^17^. TransEFVP further improves performance by combining multiple PLMs^18^. MutFormer employs a pre-trained fine-tuning paradigm to achieve high-accuracy predictions^19^. AlphaMissense integrates population frequency data and predicted structural contexts, setting a new benchmark in missense mutation pathogenicity prediction while providing predictions for all possible single amino acid substitutions in the human proteome^20^. Similarly, DeepSAV combines population and gene information, using convolutional neural networks (CNNs)^21^ to integrate sequence, structural, and functional features^22^. SuSPect, on the other hand, employs protein-protein interaction (PPI) network models to predict the pathogenicity of missense mutations effectively^23^. However, most of these methods are trained on general protein datasets, limiting their effectiveness for specific proteins like membrane proteins. Research on the pathogenicity of missense mutations in membrane proteins remains relatively sparse. Early tools such as Pred-MutHTP^6^, TMSNP^24^, mCSM-membrane^25^, and BorodaTM^26^ are developed specifically for membrane proteins but rely on shallow ML approaches. These methods do not incorporate disease-specific encoding or harness the representational power of PLMs, leaving substantial room for improvement.

Above methods for identifying pathogenic mutations provide only a binary classification, distinguishing mutations as either pathogenic or neutral. However, a more effective approach would involve further categorizing pathogenic mutations according to specific disease categories, which would enhance their clinical applicability and diagnostic value. Different diseases have distinct pathological mechanisms, and the same mutation can have varied effects across different diseases. For instance, mutations in the Cystic Fibrosis Transmembrane Conductance Regulator (CFTR) primarily cause congenital disorders of metabolism (CDM), with minimal impact on other diseases^27^. Furthermore, a single missense variant may also be responsible for the development of multiple diseases. For instance, a single missense mutation in the Proto-oncogene tyrosine-protein kinase receptor Ret can simultaneously lead to both other congenital disorders and endocrine and metabolic diseases^28,29^. This complexity underscores the need for a more granular approach to classify pathogenic mutations by disease categories. Such an approach would enable researchers to better understand the mechanisms of pathogenic mutations in different disease contexts and uncover potential connections between diseases. However, to the best of our knowledge, there are currently no prediction methods that focus on the pathogenicity of mutations associated with disease categories.

To address these challenges, we proposed MutDPAL, a DL method for predicting pathogenicity. Specifically designed for membrane proteins, MutDPAL not only classifies mutations as pathogenic or neutral, but also performs fine-grained classification to accurately predict the disease category associated with each pathogenic mutation, spanning 15 distinct categories. MutDPAL incorporates a rich set of features, including protein sequence features from ESM-1v^30^ and amino acid physicochemical and biochemical properties, transmembrane environment features derived from Biobert^31^, and disease encoding features, to construct a comprehensive, high-dimensional feature space. The model leverages multiple DL modules: a protein representation learning module, a protein-disease representation fusion module, and a transmembrane environment representation learning module, which effectively capture the complex relationships between mutations and diseases in the context of membrane proteins.

Experimental results demonstrate the superiority of MutDPAL over existing methods. In the binary pathogenicity classification task, MutDPAL achieved the highest Matthews Correlation Coefficient (MCC), outperforming current approaches by over 8%. In the multi-label disease classification, MutDPAL outperformed baseline methods across all 15 disease categories, with particularly significant improvements in digestive system diseases and other congenital disorders. Furthermore, MutDPAL’s robust performance across various protein types, including the previously unseen CFTR protein associated with CDM, highlights its generalizability and potential clinical applicability. To explore the reasons for MutDPAL’s superior performance, we conducted ablation experiments and visualization analyses. The results demonstrated that MutDPAL’s high prediction accuracy stems from leveraging pre-trained large language models (LLMs) to extract semantic information from proteins and transmembrane environment features. This is further enhanced by explicitly modeling the interaction between proteins and diseases within the context of membrane proteins. This approach significantly enhances the accuracy of missense mutation pathogenicity predictions, offering new possibilities for disease diagnosis and clinical applications.

## Results

### Overview of MutDPAL

**Fig. 1:**
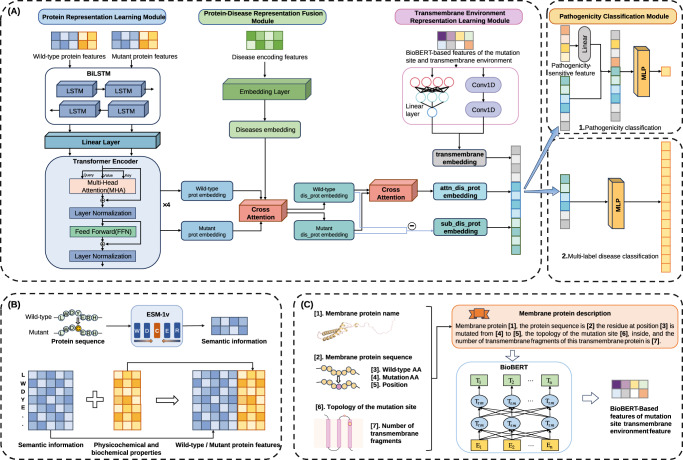
Model architecture. **A** MutDPAL mainly contains four modules: protein representation learning module, protein-disease representation fusion module, transmembrane environment representation learning module, and pathogenicity classification module. **B** The wild-type and mutant protein features. **C** BioBERT-Based features of mutation site transmembrane environment.

The architecture of MutDPAL is illustrated in Fig. 1A. We proposed a DL approach to predict the pathogenicity of missense mutations in transmembrane proteins and further classify pathogenic mutations based on disease categories.

MutDPAL consists of four key modules: (1) Protein representation learning module leveraged bidirectional long short-term memory networks (BiLSTMs) and transformer encoders to extract high-dimensional representations of wild-type and mutant protein sequences. The Semantic information was derived from ESM-1v, along with the physicochemical and biochemical properties of amino acids, as shown in Fig. 1B. (2) Protein-disease representation fusion module used a cross-attention mechanism to integrate protein and disease embeddings, enabling multimodal feature fusion and resulting in the extraction of joint protein-disease features. Here, the wild-type and mutant protein embedding were derived from the protein representation learning module, while disease embeddings were generated through disease one-hot encoding, followed by an embedding layer to produce customized representations for different diseases. (3) Transmembrane environment representation learning module employed CNNs and linear layers to learn transmembrane embedding from BioBERT-based features of mutation site’s transmembrane environment. A text-template strategy converted mutation sites and their transmembrane contexts into natural language, which were then processed with BioBERT to generate semantic features, as shown in Fig. 1C. (4) Pathogenicity classification module was responsible for determining the pathogenicity of missense mutations and performing multi-label disease classification of pathogenicity mutations, according to the contact of attn_dis_prot embedding, sub_dis_prot embedding and transmembrane embedding. These modules worked collaboratively to capture the potential impacts of mutations on transmembrane protein structure and function, providing robust support for pathogenicity prediction. While pathogenicity prediction and disease multi-label classification share the same model architecture (Fig. 1A), they differ in training objectives, label definitions, and hyperparameter settings to address the specific requirements of each task.

The data utilized in our study were derived from the MutHTP database^32^, a comprehensive repository for human transmembrane protein mutations. This database integrates mutation data from Humsavar (http://www.uniprot.org/docs/Humsavar), SwissVar^33^, 1000 Genomes^34^, COSMIC^35^, and ClinVar^36^, encompassing 183,395 pathogenic mutations and 17,827 neutral mutations in human transmembrane proteins. We constructed separate datasets from the MutHTP database to assess the pathogenicity of missense mutations in transmembrane proteins, which included the pathogenicity classification dataset (PathoClassDS) and the multi-label disease classification dataset (DiseaseClassDS). These pathogenic mutations are associated with 15 disease categories: nervous system diseases (NSD), digestive system diseases (DSD), other congenital disorders (OCD), and CDM, as well as reproductive system diseases, cardiovascular diseases (CD), respiratory diseases (RD), ISD, endocrine and metabolic diseases (EMD), musculoskeletal diseases (MD), urinary system diseases, skin diseases (SD), cancers (CN), not provided (NP), and unknown. For detailed data processing procedures, please refer to the Methods dataset section.

### MutDPAL performance in identifying general pathogenicity of missense mutations

#### Comparative analysis with SOTA methods

Assessing model accuracy on PathoClassDS with prior knowledge: In our study, we benchmarked our model against state-of-the-art (SOTA) methods on PathoClassDS, including the DL methods (MutFormer, Varipred, TransEFVP, and AlphaMissense) and shallow ML methods (Pred-MutHTP, TMSNP, mCSM-membrane, and BorodaTM). The PathoClassDS was randomly divided into training, validating, and test sets with an 8:1:1 ratio to assess the model’s accuracy on cases with prior knowledge. Performance for all methods was evaluated using the testing set of PathoClassDS. The calculation methods for all evaluation metrics are provided in Supplementary Text S1.

**Fig. 2:**
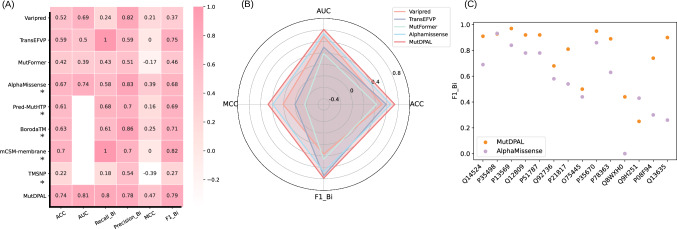
Comparison of pathogenicity classification tasks. **A** Comparison for all models, *indicates that the number of samples compared is different, the results of the comparison between MutDPAL and this method on the same number of samples are shown in Supplementary Tables S4–S8. **B** Radargram presentation of AUC, ACC, MCC, F1_Bi. **C** F1_Bi for 14 PathoClassDS proteins (UniProtIDs on the *x*-axis). The source data for this figure are available in Supplementary Data 1 (sheet 1).

Given the imbalance in data distribution, we focused on the MCC and F1_Bi (F1_score for the binary classification task), which are more appropriate evaluation metrics for imbalanced datasets. As shown in Fig. 2A and Supplementary Table S1, MutDPAL outperforms others in MCC, F1_Bi, accuracy (ACC), and area under the curve (AUC). Specifically, MutDPAL achieves an MCC of 0.47 and an F1_Bi of 0.79, surpassing the second-best performing model, AlphaMissense, by 8% and 11%, respectively. These results demonstrate the effectiveness of MutDPAL in pathogenicity prediction.

Generalization to unseen mutations via Pred-MutHTP dataset: Additionally, we conducted further experiments on the Pred-MutHTP dataset to evaluate the generalization capability of MutDPAL. The model was assessed using 10-fold cross-validation and a holdout test dataset, where mutations were grouped based on protein sequence homology clusters, ensuring that proteins in the training and validation sets were evolutionarily independent during each validation round. We compared MutDPAL with top predictors AlphaMissense and Pred-MutHTP. As shown in Table 1, MutDPAL outperforms the second-best model AlphaMissense on several key metrics. In particular, it achieved improvements of 2% in ACC, and 2% in AUC, indicating strong predictive performance even in the absence of empirical context.

**Table 1:** Comparison on unseen mutations via Pred-MutHTP dataset

Model	Validation	ACC	AUC	Recall_Bi	MCC	Specificity
Pred_MutHTP	10-fold cross	0.75	0.82	0.76	0.48	0.72
	Holdout-test	0.78	0.86	0.78	0.5	0.79
AlphaMissense	10-fold datasets^a^	0.77	0.84	0.67	**0.56**	**0.89**
	Holdout-test	0.77	0.83	0.68	0.57	**0.89**
MutDPAL	10-fold cross	**0.79**	**0.86**	**0.83**	**0.56**	0.73
	Holdout-test	**0.81**	**0.89**	**0.81**	**0.62**	0.81

The best results are indicated in bold.

The AlphaMissense data is sourced from its publicly available database of human single amino acid substitution predictions. Prediction results for each fold were extracted from the database using UniProtIDs and mutation information, and the final 10-fold results were obtained by aggregating the individual fold predictions.

^a^Indicates that 10-fold cross-validation was not performed.

#### Performance analysis based on multiple biological relevance dimensions

Performance analysis by protein type: To thoroughly evaluate the model’s performance across different protein types, we analyzed the top 15 proteins with the highest number of mutations in the test set. These proteins were selected for their representative mutation distributions and ability to reflect the model’s adaptability in complex, real-world scenarios. Although the Low-density lipoprotein receptor (LDLR) had the most samples, all of them were pathogenic mutations, making it impossible to calculate F1_Bi, thus, we will analyze it separately. For the remaining 14 proteins, we computed F1_Bi and compared them against the second model, AlphaMissense. We created a scatter plot to visually highlight these differences. As illustrated in Fig. 2C and Supplementary Table S2, our model outperforms AlphaMissense for 12 of the 14 proteins shown, achieving the highest average F1_Bi. Furthermore, among all proteins in the test set with calculable F1_Bi, MutDPAL achieves an average F1_Bi of 0.77, surpassing AlphaMissense by 19%, specific results are shown in Supplementary Table S3. Notably, for proteins such as patched homolog 1, Fibrocystin, and Nesprin-2, our model improves upon the second-best model by 64%, 44%, and 44%, respectively. This demonstrates that our model effectively distinguishes the pathogenicity of different mutations within the same membrane protein. Further analysis reveals that 97% of LDLR mutations were classified as pathogenic, and our model accurately identified all positive samples, outperforming AlphaMissense by 31%. This demonstrates the model’s ability to effectively capture pathogenic features and achieve precise classification, even for proteins with extreme sample distributions and functional complexities.

Nesprin-2 is a structurally complex transmembrane protein containing multiple highly conserved spectrin repeat domains^37^. These domains are crucial for maintaining the connection between the nucleus and the cytoskeleton and are evolutionarily preserved^38^. This conservation enables sequence-based features to effectively capture functional information. MutDPAL leverages its protein representation learning module to extract high-dimensional embeddings from ESM-1v semantic representations and physicochemical features of amino acids. This enables the model to effectively capture inherent sequence patterns and physicochemical properties within spectrin repeats, ASH domains, and CH domains. Specifically, the BiLSTM component captures local contextual relationships within the sequence, while the Transformer Encoder learns long-range dependencies, implicitly reflecting the overall domain organization. This allows the model to identify subtle yet functionally significant changes caused by mutations, ultimately enhancing its pathogenicity prediction performance. In contrast, Cadherin-23 is predominantly found in inner ear hair cells, where its function often depends on specific cellular contexts and dynamic regulation^39^. These factors may be difficult to fully capture using static sequence features alone, leading to relatively poor predictive performance of models for this protein.

Performance analysis across protein functional categories: To evaluate the performance of MutDPAL more comprehensively, we further analyzed its prediction performance on different functional categories of proteins. We divided transmembrane proteins into six categories: G protein-coupled receptor (GPCR), ion channel, receptor, transporter, kinase and other, and evaluated the prediction performance of the model in each of these categories. As shown in Table 2, MutDPAL has high F1_Bi scores in all protein function categories (all above 0.73). Among them, the highest F1_Bi (0.83) was found in the GPCR structure with an AUC of 0.93, while the F1_Bi of ion channels, receptors, transporter proteins, and kinases ranged from 0.80 to 0.83, suggesting that MutDPAL has a strong generalization ability in the mutation prediction task across different protein function categories.

**Table 2:** Performance of MutDPAL on different protein function categories

Model	ACC	AUC	Recall_Bi	Precision_Bi	MCC	F1_Bi
GPCR	**0.88**	**0.93**	0.83	**0.83**	**0.74**	**0.83**
Ion channel	0.74	0.74	**0.86**	0.80	0.32	**0.83**
receptor	0.74	0.79	0.82	0.79	0.42	0.80
Transporter	0.76	0.80	0.85	0.79	0.44	0.82
kinase	0.74	0.80	0.84	0.74	0.46	0.79
other	0.72	0.80	0.71	0.74	0.44	0.73

The best results are indicated in bold, Recall_Bi denotes the Recall value for binary classification and Precision_Bi denotes the Precision value for binary classification.

Performance analysis across different membrane topological regions: To evaluate the mutation prediction performance of MutDPAL across different membrane topology regions, we categorized mutations based on the protein’s membrane topology into cytoplasmic, transmembrane, and extracellular regions, and separately analyzed the prediction results for each region. As shown in Supplementary Fig. S1, MutDPAL achieves superior AUC performance compared to other methods across all regions. Moreover, the comparative analysis of MCC further demonstrates MutDPAL’s consistent superiority across different membrane topological regions. In particular, MCC in the transmembrane, cytoplasmic, and extracellular regions were 1%, 7%, and 10% higher than the suboptimal model, respectively (For details, see Supplementary Tables S9–S11). This result is consistent with the performance trend of the overall dataset, indicating that MutDPAL can consistently maintain its advantages in different membrane topological regions.

### MutDPAL performance in identifying disease classes of pathogenic missense mutations

Our approach is the first to classify pathogenic mutations into distinct disease categories, specifically in the context of membrane proteins. To evaluate its performance, we compared it with several traditional multi-label classification methods and enhanced DL approaches. For traditional ML models, we utilized DecisionTree (DT)^40^, Extremely Randomized Trees (ExtraTree)^41^, Extremely Randomized Trees (ExtraTrees)^41^, K Nearest Neighbors (KNN)^42^, MLP, and RF^43^, ensuring a fair comparison by using input features consistent with those of our model. For DL methods, we adapted existing models originally designed for binary pathogenicity classification tasks (Varipred, TransEFVP, and MutFormer) by modifying their output layers to support multi-label classification. DiseaseClassDS was randomly split into train, valid, and test sets in an 8:1:1 ratio. All methods were evaluated on the test set using standard multi-label classification metrics to ensure a comprehensive performance comparison. Details of the calculation methods for these metrics can be found in Supplementary Text S2.

#### Overall performance of MutDPAL on DiseaseClassDS

First, we evaluated the effectiveness of our method in labeling pathogenic mutations across 15 disease categories. As indicated in Table 3, our model outperforms other methods across all four multi-label evaluation metrics. It achieves a Precision_ML of 0.78, Recall_ML of 0.76, F1_ML of 0.76, and Hamming Loss of 0.03, surpassing the second-best model, MLPClassifier, by 9%, 10%, 11%, and 1%, respectively. These results demonstrate the significant advantage of our model in handling the complex task of classifying pathogenic mutations in membrane proteins. Additionally, MLPClassifier ranked second overall, validating the effectiveness of the input features.

**Table 3:** Comparison in terms of multi-label metrics

Model	Precision_ML(↑)	Recall_ML(↑)	F1_ML(↑)	Hamming Loss(↓)
DT	0.54	0.61	0.55	0.07
ET	0.5	0.57	0.51	0.07
ETs	0.55	0.58	0.55	0.04
KNN	0.51	0.52	0.51	0.05
MLP	0.69	0.66	0.65	0.04
RF	0.52	0.55	0.53	0.04
Varipred	0.41	0.39	0.39	0.06
TransEFVP	0	0	0	0.08
MutFormer	0.41	0.38	0.39	0.06
MutDPAL	**0.78**	**0.76**	**0.76**	**0.03**

The best results are indicated in bold, Recall_ML denotes the Recall score for the multi-label classification task, Precision_ML denotes the Precision score for the multi-label classification task and F1_ML denotes the F1_score for the multi-label classification task.

#### Assessing pathogenic missense mutations based on disease categories

**Fig. 3:**
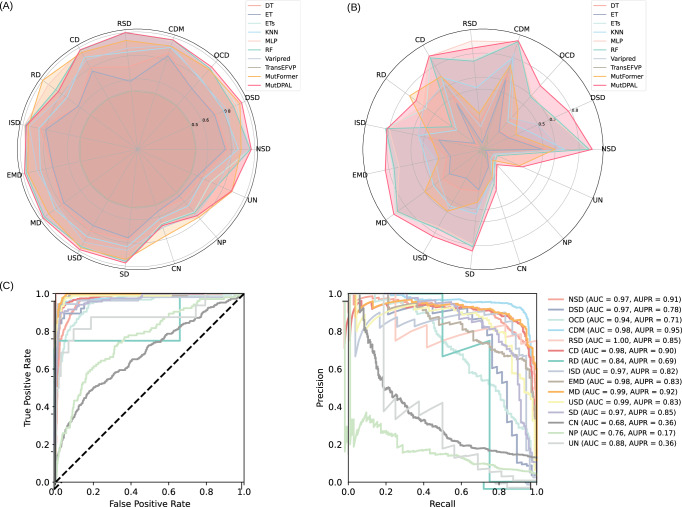
Comparison of AUC and AUPR by disease. **A** Comparison of AUC for 15 disease categories. **B** Comparison of AUPR for 15 disease categories. **C** Categorization of the 15 diseases in MutDPAL. The source data for this figure are available in Supplementary Data 1 (sheet 2).

To evaluate the model’s performance in binary classification for individual diseases, we selected AUC and Area Under the Precision versus Recall Curve (AUPR) as key metrics and visualized the results. When calculating AUC and AUPR, negative samples for each disease were defined as those not associated with that specific disease. For example, if NSD is the first target disease, a positive sample is (1, B, B, B, …), and a negative sample is (0, B, B, B, …), where B represents the binary labels (0 or 1) for other diseases. As illustrated in Fig. 3B, our model achieved the highest AUPR scores in 11 of the 15 disease categories. Notably, it excels in the DSD and OCD, outperforming the second-best model by 12.64% and 10.51%, respectively, showcasing its strong feature-learning capability for these specific categories. For the four categories where our model did not achieve the highest performance, we observe that two had relatively small sample sizes (34 and 67 samples), which likely limited the model’s ability to extract meaningful features. This highlights that while MutDPAL effectively captures key characteristics in larger datasets, its performance can be constrained in categories with very limited data. Detailed scores and comparative results are provided in Supplementary Tables S12 and S13. In addition, we have also tried to include neutral samples as a control, and the experimental results are presented in Supplementary Table S14.

Furthermore, we used Receiver Operating Characteristic Curve (ROC curve) and Precision-Recall Curve (PR curve) to evaluate the performance of MutDPAL across 15 disease classification tasks. As shown in Fig. 3C, ROC curves closer to the top-left corner indicate higher sensitivity and lower false positive rates for a given class, while PR curves closer to the top-right corner suggest better precision and recall. The results demonstrate that our model performs exceptionally well in classifying MD (AUC = 0.99, AUPR = 0.92) and CDM (AUC = 0.98, AUPR = 0.94). This superior performance is likely due to the higher separability of these classes in the feature space. In addition, MutDPAL also shows strong classification performance for NSD and SD, with AUC values exceeding 0.97 for both categories.

While MutDPAL demonstrates robust classification performance across most disease categories, its predictions may be less stable for diseases with fewer samples due to data scarcity. For example, in the DSD category, the model achieved an AUC of 0.97 and an AUPR of 0.78, slightly lower than categories with larger sample sizes. To assess the predictive reliability of MutDPAL in small-sample categories, we performed bootstrap resampling (1000 iterations) for these categories (e.g., DSD) in the test set. Ultimately, the 95% confidence interval (CI) for its AUPR was [0.653, 0.892], which was wider compared to the interval for high-sample categories (e.g., NSD, AUPR CI = [0.888, 0.929]) but still significantly higher than the randomized classification level (AUC = 0.5, *p* < 0.01). Overall, our model demonstrates outstanding accuracy and stability in multi-label classification of membrane protein mutations. However, there remains room for improvement when addressing categories with dispersed feature distributions or fewer samples.

#### Case study: analyzing mutations labeled with a single disease

In the DiseaseClassDS, 89% of mutations are single-label cases, which means that each mutation causes only a single disease. We performed a separate analysis for these cases, as shown in Supplementary Table S15. It is evident that MutDPAL significantly outperforms other models in single-label prediction. It achieves Precision_ML, Recall_ML, F1_ML, and HammingLoss scores of 0.77, 0.80, 0.78, and 0.03, respectively, underscoring the model’s robust performance in single-label prediction.

**Fig. 4:**
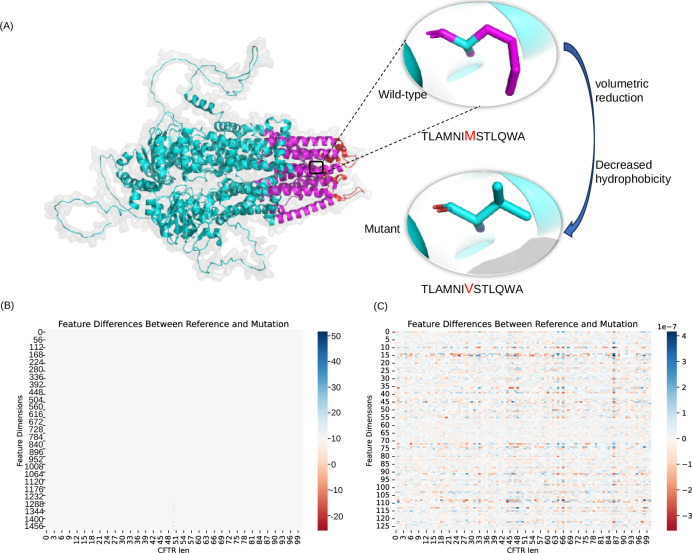
Case study. **A** CFTR mutant structure display, colored according to the topology of the residues, green for cytoplasmic, purple for transmembrane, red for extracellular. **B** Input features before protein representation learning module (Wild-type protein features vs. Mutant protein features). **C** Output features after protein representation learning module (Wild-type prot embedding VS Mutant prot embedding), CFTR len indicates the length of the CFTR protein, here 101. The source data for this figure are available in Supplementary Data 2.

To validate the effectiveness of MutDPAL in addressing real-world disease mutations, we selected CDM as a representative case study. This case examines a known missense mutation in the CFTR (Fig. 4). This mutation replaces methionine (M) with valine (V) in the wild-type protein, causing subtle changes in local polarity and hydrophobicity. These alterations may disrupt hydrophobic interactions and van der Waals forces between amino acid side chains, affecting protein folding stability and, in turn, the function of the transmembrane channel. To demonstrate MutDPAL’s capability in characterizing this mutation, we analyzed the feature distributions of both the wild-type and mutant CFTR proteins before and after model training. As shown in the heatmap (Fig. 4B, C), the darker the color, the greater the difference in features before and after the mutation. The results reveal that prior to training, the differences in the high-dimensional feature space between the wild-type and mutant proteins were minimal, and the feature changes caused by the mutation are more insidious and difficult to distinguish directly. However, after processing through the protein representation learning module, the feature differences before and after the mutation are significantly enhanced, resulting in a significantly higher differentiation in the low-dimensional embedding space. This result not only validates the effectiveness of the protein representation learning module of MutDPAL, but also further improves the accuracy of disease classification. Notably, this mutation has been biologically validated and is strongly linked to the development of CDM^44^. We also examined other mutations in the test set and validated them against existing literature. For example, a missense mutation in the Activin receptor type-1-like has been confirmed to correlate with CD^45^, while a missense mutation in Phosphatidylcholine translocator ABCB4 is linked to DSD^46^. These findings provide robust biological support for the model’s predictions.

In addition, we also explored the potential predictive power of MutDPAL in a single disease class, exemplified by a mutation in ATP-binding cassette sub-family C member 8 (ABCC8). This mutation was originally annotated in DiseaseClassDS as “familial hyperinsulinemic hypoglycaemia 1”, a congenital disorders of metabolism^47^ (889, K to T). MutDPAL predicts that this mutation is not only associated with CDM, but also with EMD. By reviewing the UniProt variant entries and KEGG disease classifications, we found that this variant is indeed also associated with Permanent neonatal diabetes mellitus, monogenic diabetes, and Type 2 diabetes mellitus, all of which are EMD. This finding suggests that certain mutations may affect multiple disease categories, even when initially classified as a single disease mutation, and that MutDPAL has the potential to reveal such hidden disease associations.

#### Case study: analyzing the same mutation across different diseases

In the DiseaseClassDS, 11% of mutations are multi-label cases. We performed a separate analysis for this multi-label cases, as shown in Supplementary Table S16. It is evident that MutDPAL significantly outperforms other models in multi-label prediction. It achieves a Precision_ML of 0.86, Recall_ML of 0.51, F1_ML of 0.62, and Hamming Loss of 0.08, surpassing the second-best model, MLPClassifier, by 8%, 5%, 6%, and 1%, respectively, highlighting its ability to effectively capture complex relationships between multiple disease labels.

To more visually illustrate the predicted performance of the same mutation in different disease contexts, we selected a missense mutation in transient receptor potential vanilla subfamily V member 4 (TRPV4) as a case study (797, E to K). The mutation significantly alters the bulk and charge properties of the amino acid, further affecting the structure and function of the protein. Notably, this mutation has been reported to be associated with both MD and NSD^48–50^, and MutDPAL was able to accurately predict its association with both types of disorders. In addition, we further retrieved other multilabel mutations from the test set and validated their predictions against MutDPAL. For example, the missense mutation (143, S to F) in Epithelial sodium channel subunit beta (SCNN1B) was documented as associated with RD and EMD in the test set. As another example, the missense mutation (244, T to I) in Interleukin-7 receptor subunit alpha (IL7R) was recorded as associated with CN and ISD. Meanwhile, MutDPAL provided the same predictive results for both of these mutations. These results further validate the effectiveness of the MutDPAL model in practical applications.

#### Analyzing potential associations among different disease categories

**Fig. 5:**
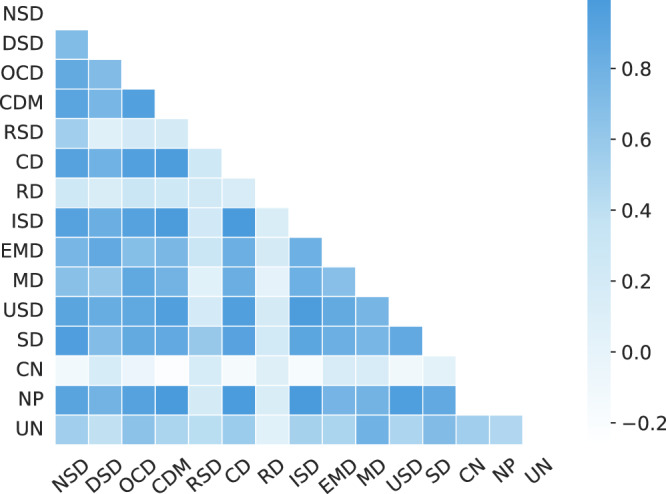
Correlation statistics between the 15 diseases, where color shade is directly proportional to the correlation between the two diseases. The source data for this figure are available in Supplementary Data 1 (sheet 3).

Mutations in membrane proteins can lead to two or more categories of diseases. To illustrate the relationships between different diseases, we constructed a co-occurrence matrix of 15 disease categories and visualized it as a heatmap. Specifically, the correlation between any two diseases was quantified by calculating the probability of their co-occurrence in the same mutation. As shown in Fig. 5, certain diseases exhibit strong positive correlations, such as CDM and OCD, ISD and CD, and NSD and MD. These correlations may stem from shared metabolic pathways or immune responses, leading to similar pathogenic effects across multiple systems. For instance, studies suggest that CDM and OCD share pathogenic effects through the pyruvate metabolism pathway^51^, while ISD and CD are linked through inflammatory factor interactions^52^. These multi-label cases are well represented in our newly developed DiseaseClassDS. For example, the observed correlation between NSD and MD is supported by research showing that a missense mutation in the Transient Receptor Potential Cation Channel Subfamily V Member 4 can cause both types of diseases^49,50^. This indicates potential similarities in their pathogenic mechanisms.

Additionally, cancer shows weaker correlations with most other disease categories. This may be attributed to the high heterogeneity of cancer mutations and their distinct pathogenic mechanisms, which often involve mutations in tumor suppressor genes (e.g., TP53 and RB1) and changes in the tumor microenvironment^53,54^. Unlike other diseases, cancer mutations primarily affect pathways related to cell proliferation and apoptosis, rather than cross-system metabolic or immune response pathways^55^. This likely explains the weaker correlations and provides important directions for investigating the effects of cancer-specific mutations.

#### Analysis of module contributions and representation importance

To understand the reasons for MutDPAL’s superior performance, we conducted ablation experiments and visualization analysis, revealing the following key insights: First, the protein representation learning module amplified the differences in sequence features caused by mutations, enabling the model to better distinguish between wild-type and mutant protein features. Second, the cross-attention mechanism between protein and disease features effectively combined both feature sets, significantly improving the model’s accuracy in disease prediction tasks. The learning of transmembrane environmental features further enabled the model to capture the structural and functional characteristics of membrane proteins, improving prediction accuracy. Finally, dimensionality reduction and visualization results validated MutDPAL’s effectiveness in identifying pathogenic mutations in membrane proteins and performing multi-label disease classification. Specifically, after DL, pathogenic and neutral samples displayed clear classification boundaries in the feature space, with samples from different disease categories clustering into distinct groups, significantly reducing overlap.

#### Ablation study to evaluation of each module’s contribution in MutDPAL

**Fig. 6:**
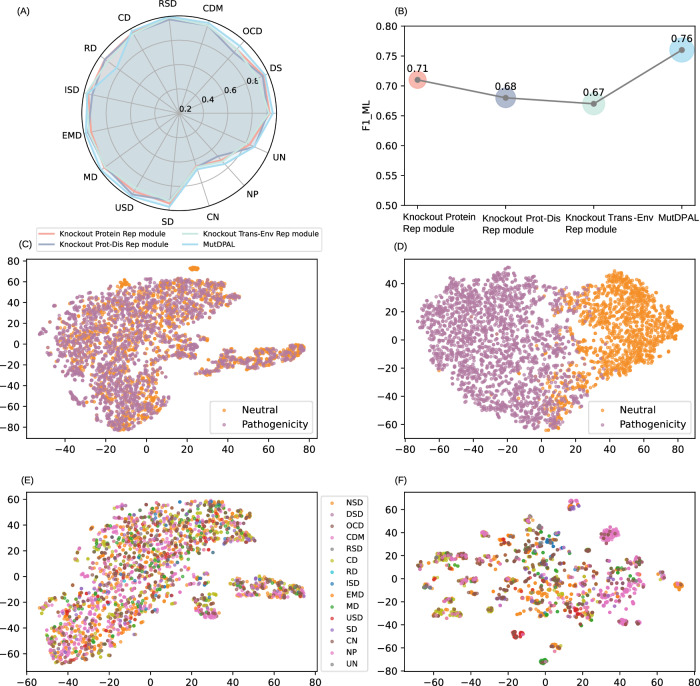
Ablation experiments and visualization. **A** Comparison of ablation experiments on individual diseases. **B** Comparison of ablation experiments on overall F1_ML. Knockout Protein Res module represents the removal of the protein representation learning module, Knockout Prot-Dis Rep module represents the removal of the protein-disease representation fusion module, and Knockout Trans-Env Rep module represents the removal of the transmembrane environment representation learning module. **C** Input raw feature space for neutral and pathogenic mutations. **D** Trained feature space for neutral and pathogenic mutations. **E** Input raw feature space for pathogenic mutations of 15 diseases. **F** Trained feature space for pathogenic mutations of 15 diseases. The source data for this figure are available in Supplementary Data 3.

To evaluate the necessity of each module within MutDPAL, we conducted ablation experiments. MutDPAL consists of three primary components: a protein representation learning module, a protein-disease representation fusion module, and a transmembrane environment representation learning module. In the ablation experiments, we removed one module at a time and retrained the model to assess its contribution to overall performance. These experiments were performed on the multi-label disease classification task. The results are shown in Fig. 6A, B, indicating that each module contributes to the final performance to varying degrees. Notably, the protein-disease representation fusion module and the transmembrane environment representation learning module significantly enhanced the F1_ML. Removing the protein-disease representation fusion module led to a drop in F1_ML from 0.76 to 0.68, while removing the transmembrane environment representation learning module reduced F1_ML from 0.76 to 0.67, resulting in performance decreases of 8% and 9%, respectively. In contrast, removing the protein representation learning module resulted in only a decrease in F1_ML from 0.76 to 0.71. An important reason for this small decrease is that even with the removal of this module, the protein representation information is still retained to some extent through the other modules. The removal of the protein-disease representation fusion module and the transmembrane environment representation learning module, on the other hand, directly loses the integrated disease and membrane protein-specific features, respectively, and the lack of information is more pronounced, thus leading to a larger decrease in the performance. These findings highlight the importance of module synergy in MutDPAL. By effectively capturing protein and disease multimodal features and transmembrane environment feature, the model demonstrates superior performance in multi-label disease classification tasks.

#### Visualizing the importance of learned representations for missense mutations, transmembrane proteins and diseases

To assess MutDPAL’s ability to extract high-level features from input data, we used t-SNE^56^ to visualize the initial input raw features and the final trained feature representations on the test set. As shown in Fig. 6C–F, the input raw features, after t-SNE dimensionality reduction, exhibit a relatively uniform distribution and lack distinct clustering. This indicates weak discriminative power in their raw form. After MutDPAL’s feature learning modules, these features were mapped into a more discriminative feature space. Specifically, for pathogenicity classification, samples representing pathogenic and neutral categories displayed clear boundaries in the t-SNE visualization. Similarly, in the multi-label disease classification task, samples from different disease categories formed relatively distinct clusters, with significantly reduced overlap between categories. These results demonstrate that the model effectively captures deep information from the input features, enhancing differentiation among disease categories in the feature space.

## Discussion

Mutations in membrane proteins are closely linked to various genetic disorders, making them a critical focus for understanding disease mechanisms. In this study, we propose an innovative DL approach, MutDPAL. The method first distinguishes pathogenic mutations from neutral ones in membrane proteins and, for the first time, further classifies pathogenic mutations into 15 specific disease categories. To train the model, we curated a novel dataset derived from the specialized membrane protein database MutHTP, covering 15 distinct disease categories. MutDPAL incorporates a multitude of features, including protein sequence semantic information extracted via ESM-1v, the physicochemical and biochemical properties of amino acids, transmembrane environment features generated based on text-template strategy, and disease coding features, to construct a comprehensive, high-dimensional representation. MutDPAL employs distinct DL modules to learn complex feature representations tailored to each feature type. These include a protein representation learning module, a protein-disease representation fusion module, and a transmembrane environment representation learning module.

We conducted a comprehensive evaluation of MutDPAL. In the binary pathogenicity classification task, MutDPAL achieved a MCC of 0.45, representing a performance improvement of over 6% compared to current approaches. Further analysis of the model’s F1_Bi performance across different protein types revealed that MutDPAL’s average F1_Bi was 0.72, which was 17% higher than the next best model, Alphamissense. Especially for patched homolog 1, Fibrocystin and Nesprin-2, F1_Bi of MutDPAL is improved by 18%, 16%, and 15% respectively, highlighting its applicability and robustness across different protein types. Additionally, we explored MutDPAL’s performance in the multi-label disease classification task. The results show that MutDPAL’s prediction ability is equally impressive across 15 disease categories, with an F1_ML of 0.76, outperforming all baseline methods. Specifically, MutDPAL’s performance is particularly noteworthy in DSD and OCD, with AUPR being 12.64% and 10.51% higher than the next best model, respectively. To further validate the model’s applicability in real-world scenarios, we conducted a case study to assess its ability to predict pathogenicity for unseen proteins. Taking a known pathogenicity mutation in CFTR as an example, MutDPAL accurately captured and highlighted the feature changes caused by the mutation and successfully identified the associated disease category, providing strong biological support for the model. Notably, MutDPAL does not rely on the outputs of other pathogenicity prediction tools, ensuring the model’s independence and robustness.

To understand the factors driving MutDPAL’s superior performance, we conducted ablation studies and visualization analyses. It’s clear that MutDPAL’s accurate predictions are primarily attributed to its focus on Disease-Protein Association Learning in the context of membrane proteins, which is reflected both in its feature representation and model architecture design. From the perspective of feature representation, MutDPAL is the first model to use the pre-trained LLM BioBERT for representing transmembrane environment features in the prediction of missense mutations in membrane proteins. By extracting the transmembrane environment features and integrating them with protein features and disease features, the model effectively captures the relationship between mutations and specific diseases. The protein features incorporate not only traditional physicochemical and biochemical properties of amino acids, but also rich semantic information extracted from sequences using ESM-1v, which results in a high-dimensional representation of missense mutations. From the perspective of model architecture design, MutDPAL first employs BiLSTMs to capture local contextual information within the sequence of membrane protein features. These features are then dimensionality-reduced using a linear layer and passed into a Transformer encoder, which utilizes a multi-head self-attention mechanism to capture global dependencies from various perspectives across the protein sequence. This helps the model to comprehensively understand the structural and functional changes in the protein both before and after mutation. Importantly, MutDPAL employs a cross-attention mechanism to merge wild-type and mutant protein embeddings with disease-specific embeddings, capturing the interdependencies between diseases and mutations. This fusion allows for a deeper understanding of how specific mutations affect disease outcomes, further enhancing the model’s predictive accuracy.

Although our method excels in both overall pathogenicity prediction for membrane proteins and multi-label disease classification, there is still room for improvement in terms of prediction accuracy for some specific diseases (e.g., cancer) as well as for some specific proteins (e.g., Cadherin-23). In the future, we plan to expand the disease categories with small samples, alleviate the data scarcity problem using methods such as migration learning, and introduce more contextual information, such as PPI networks, tissue-specific expression data, and dynamic structural predictions from AlphaFold, to enhance feature encoding. In addition, we will explore more complex disease coding approaches and further improve the accuracy of MutDPAL for pathogenicity prediction and disease classification tasks through multimodal data fusion. Moreover, since MutDPAL’s input includes transmembrane protein-related attributes (e.g., the topological structure of mutation sites and the number of transmembrane segments), its direct application to non-transmembrane proteins may require further adaptation and feature integration. We plan to integrate more features (e.g., structural and evolutionary information) applicable to non-membrane proteins as well as other proteins to extend the model to a wider range of protein types. In summary, MutDPAL has made significant strides in the pathogenicity prediction of missense mutations in membrane proteins and multi-label disease classification, offering substantial potential for supporting clinical diagnosis.

## Methods

### Overview of datasets

To evaluate the pathogenicity of missense mutations in membrane proteins and determine their associated disease categories, we constructed two distinct datasets for pathogenicity classification and multi-label disease classification, utilizing data from the MutHTP database. MutHTP integrates multiple sources, including Humsavar, SwissVar^33^, 1000 Genomes^34^, COSMIC^35^, and ClinVar^36^, with a primary focus on curating and categorizing transmembrane protein mutations. As depicted in Supplementary Fig. S2 the original MutHTP dataset exhibits a pronounced bias toward cancer-related mutations, with 165,293 entries accounting for approximately 85.9% of all disease classes.

### Pathogenicity classification dataset

**Fig. 7:**
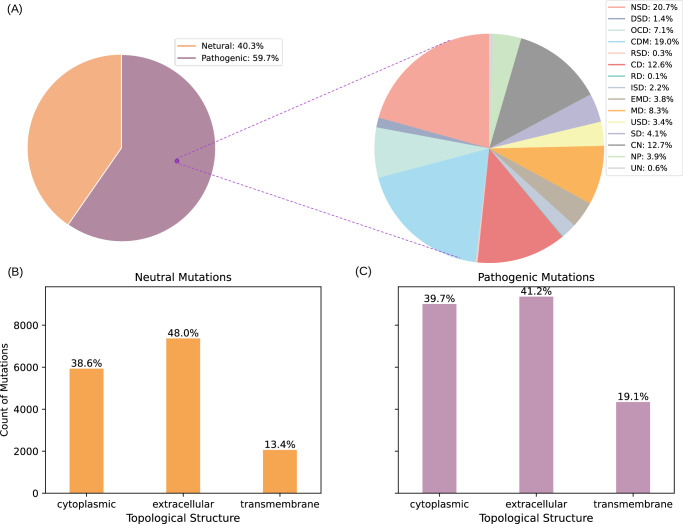
Data statistics. **A** Percentage of PathoClassDS and DiseaseClassDS. **B** Topological distribution of mutation sites. **C** Topological statistics in 15 diseases. It is important to note that the starting point of each section is not zero, but is stacked from the top of the next section. For example, the value of the bottom yellow section starts at zero, while the purple section starts stacking from the top position of the yellow section, and so on. The total height represents the cumulative sum of the values of each section. The source data for this figure are available in Supplementary Data 1 (sheet 4).

PathoClassDS: To construct the dataset for pathogenicity classification, we categorized missense mutations from the filtered MutHTP database into two groups: pathogenic (labeled as 1) and neutral (labeled as 0), based on the dis_class label. To ensure data integrity, we excluded entries where the wild-type residue did not match the reference sequence at the mutation site or where the mutation position exceeded the sequence length. Given the inherent imbalance caused by the overrepresentation of cancer-related mutations, we retained only those mutations that were present in at least two of the five databases (Humsavar, SwissVar^33^, 1000 Genomes^34^, COSMIC^35^, and ClinVar^36^). After filtering, the final PathoClassDS dataset comprised 38,063 mutation entries, including 22,708 pathogenic and 15,355 neutral mutations. The distribution of the final filtered dataset is illustrated in Fig. 7A.

To evaluate the model’s predictive performance, we employed a random data-splitting strategy, dividing the dataset into training, validation, and test sets in an 8:1:1 ratio. This approach facilitates the assessment of the model’s accuracy in handling cases with empirical data. Some example data can be found in Supplementary Table S17. Pred-MutHTP dataset: To further assess the generalization ability of our model under more stringent conditions, we utilized a non-redundant dataset, Pred-MutHTP^6^. This dataset was constructed by clustering proteins based on sequence identity (threshold >40%) using the CD-HIT tool. Within each cluster, only representative proteins and all their associated mutations were retained. For non-representative sequences, we retained only single mutation records at conserved positions, determined by conservation analysis of the mutation sites. Additionally, for mutations occurring at conserved positions, we further refined the dataset by considering amino acid substitution types (e.g., A → T, G → S) to eliminate redundant entries with identical substitutions at the same conserved sites. As a result, the Pred-MutHTP dataset consists of 11,846 disease-causing mutations and 9533 neutral mutations.

For this dataset, we adopted a strict data partitioning strategy similar to that of Pred-MutHTP: mutations were grouped based on protein sequence identity clustering, ensuring that proteins in the training and validation sets were evolutionarily independent in each iteration of cross-validation. Specifically, we performed 10-fold cross-validation, treating each cluster as an independent group, and tested the model on a holdout test dataset (20%), which is also evolutionarily independent of the training set, thereby evaluating the model’s predictive performance on unseen data in a more rigorous setting.

### Disease classification dataset

The pathogenic data in the MutHTP database covers 15 disease categories. All pathogenic missense mutations from PathoClassDS were selected to create a multi-label dataset named DiseaseClassDS, based on disease classification. This means that a single missense mutation may be associated with multiple diseases, such as NSD and MD. The distribution of classes in DiseaseClassDS is illustrated in Fig. 7A.

Compared to the highly skewed cancer-dominant distribution in the original MutHTP dataset, the filtered DiseaseClassDS exhibits a more balanced representation across different disease categories, effectively mitigating data imbalance to some extent. For detailed information on the 15 disease categories, their corresponding sample counts, and the distribution across the training, validation, and test sets, please refer to Supplementary Table S18.

### Topological distribution of mutations in membrane proteins

Transmembrane segments are often essential for membrane proteins to perform their functions, and the topological location of mutation sites can directly impact the stability and function of the protein^57^. Analyzing the topological distribution of these mutation sites provides valuable insights into how mutations influence membrane protein behavior. Considering the inherent flexibility of transmembrane protein structures and the absence of some protein structural, we have chosen to focus solely on their topology for the current analysis. As some membrane proteins lack natural topological structures, the TMbed tool was employed to predict their topological information based on protein sequences^58^. The predictions yielded five types of topological structures: transmembrane beta strand (B), transmembrane alpha helix (H), signal peptide (S), non-transmembrane, inside (i), and non-transmembrane, outside (o). We grouped these five types into three categories: transmembrane (including H and B), cytoplasmic (i), and extracellular (including o and S, the latter usually located at the N-terminus of newly synthesized proteins and excised in mature proteins^59^).

We performed a detailed statistical analysis on data from both PathoClassDS and DiseaseClassDS, as shown in Fig. 7B, C. In the topology of membrane proteins, transmembrane regions are embedded directly within the lipid bilayer. These regions play a crucial role in maintaining protein structural stability, facilitating ligand binding, and ensuring efficient signal transduction. Mutations in transmembrane regions can lead to protein instability and functional loss, exhibiting a higher pathogenic potential^60^ (13.4% for neutral mutations vs. 19.1% for pathogenic mutations). In contrast, non-transmembrane regions are largely dependent on water-mediated interactions, which makes them often more sensitive and susceptible to external influences in response to environmental changes such as pH fluctuations, post-translational modifications or protease degradation^61^. These findings suggest that protein topology may play a critical role in disease pathogenesis.

### Overview of feature extaction

MutDPAL integrated three key types of features: wild-type and mutant protein features, BioBERT-based features of the mutation site and transmembrane environment, and disease encoding features. To characterize wild-type and mutant proteins, we extract sequence-based features through two complementary approaches: (i) a DL-based semantic representation of protein sequences (e.g., using pretrained language models), which captures evolutionary and functional patterns; (ii) explicit integration of physicochemical and biochemical properties of amino acids (e.g., hydrophobicity, charge, and polarity). This dual strategy enables a comprehensive quantification of structural and functional changes induced by mutations. To address the structural complexity of transmembrane proteins, a text-template strategy was employed to convert the mutation sites and their transmembrane environmental context into natural language descriptions. These descriptions were then processed by BioBERT to generate semantic features, capturing the deeper impact of mutations on membrane protein functionality. For disease information, one-hot encoding was used to convert different disease categories into vector representations^62^, eliminating any implicit numerical or ordinal relationships between categories. This approach ensures that the features are independently and accurately represented in both binary pathogenicity classification and multi-label disease classification tasks.

### The wild-type and mutant protein features

The wild-type and mutant protein features: To standardize the input sequence length and focus the task on the mutated regions, we truncated each protein sequence to 101 residues, positioning the mutation site at the center. We extracted semantic information as well as physicochemical and biochemical properties of amino acids for wild-type and mutant proteins, respectively, based on the protein sequence. First, we generated semantic information of protein sequences using ESM-1v, a model based on protein language modelling and trained via large-scale unsupervised learning, which captures the semantic relationships between residues. For each protein sequence, ESM-1v ultimately represents it as a 101 × 1280 feature matrix. If there are fewer than 50 residues on either side of the mutation site, we pad the sequence with zeros to ensure consistent dimensionality of the feature matrix.

There are significant differences in the physicochemical and biochemical properties among different amino acids. Missense mutations not only lead to changes in the properties of the amino acids themselves, but may also affect their interactions with surrounding amino acids, potentially affecting the function of the protein. Therefore, we collected 213 physicochemical and biochemical properties of 20 amino acids from the AAIndex1 database, reflecting missense mutations’ effects on protein function^63^. The AAIndex1 database contains 566 physicochemical properties of 20 amino acids and provides correlation information between the properties. However, as there may be a high correlation between some attributes, for example, the correlation between alpha-CH and alpha-NH chemical shifts is 0.949, we filtered them using the correlation information provided by AAIndex1 and retained only the first occurring attributes to reduce redundancy of information. The final screening yielded 213 attributes (The detailed criteria are provided in Supplementary Text S3). For protein sequences with fewer than 50 residues at either end, we padded them with zeros, resulting in a 101 × 213 feature matrix.

Finally, we concatenated the semantic information of ESM-1v with the physicochemical and biochemical features to characterize the wild-type and mutant proteins as 101 × 1493 dimensional feature matrices (see Fig. 1B). Such concatenation can combine the advantages of semantic information and physicochemical and biochemical properties to help the model more comprehensively capture the multi-level effects of mutations on protein structure and function.

### BioBERT-Based features of mutation site’s transmembrane environment

BioBERT is a DL model based on the Bidirectional Encoder Representations from Transformers architecture, designed for the biomedical domain, which uses a large biomedical corpus for pre-training and performs well on biomedical text processing tasks. Membrane proteins have unique biological functions and structural properties, such as the number of transmembrane fragments and the topology of residues, and thus the study of their mutational effects requires specialized methods for feature extraction. In this study, we adopt a text-template strategy to convert the transmembrane information of membrane protein mutation sites into natural language descriptions^64^. This approach integrates the structural information and mutation features of membrane proteins and provides input for BioBERT to generate high quality semantic representations. For example, given a feature vector (e.g., “membrane protein name”, “protein sequence”, “mutation site”, “wild-type residues”, “mutant residue“, “mutation site topology”, “number of transmembrane fragments”) and their values (e.g., “cystic fibrosis transmembrane conductance regulator”, “MQRSP……”, “551”, “G”, “D”, “non-transmembrane, inside”, 12’), we transform the examples into the following natural language description: ‘Transmembrane protein CFTR, the protein sequence is MQRSP……, the residue at position 551 is mutated from G to D, the topology of the mutation site is non-transmembrane, inside, and the number of transmembrane fragments of this transmembrane protein is 12’. This transformation can effectively retain key information such as the number of transmembrane fragments, topological features of the mutation site, etc., which are processed by BioBERT to generate 768-dimensional feature vectors. BioBERT is able to adequately capture subtle differences in semantic context by pre-training on a large amount of data from biomedical fields. Based on this representation, we further analyze the potential impact of mutations on membrane protein functions, significantly improving the accuracy of mutation effect prediction.

### Disease encoding features

To represent disease information, we used one-hot encoding to encode different disease categories. This approach provides a clear translation of disease categories into encoded vectors, thereby avoiding the potential interference of sequential relationships or numerical differences between categories in model learning. In the PathoClassDS, diseases are categorized into two classes: Pathogenic and Neutral. These categories are encoded as 2-dimensional vectors, where pathogenic represented as [1,0] and neutral as [0, 1]. In the DiseaseClassDS, the same encoding method is applied, but each disease category is mapped to a 15-dimensional one-hot vector. In this representation, each dimension corresponds to a specific disease category, with the position representing the target disease is set to 1, and all other positions are 0.

### Pathogenicity-sensitive features

#### To enhance the characterization of pathogenicity feature differences, we incorporated additional features into the pathogenicity prediction task

AAIndex representation: We extracted three sets of amino acid index features: (1) a 213-dimensional difference between wild-type and mutant residues from AAIndex1. (2) the corresponding 94 substitution matrix scores from AAIndex2^63^. (3) the 47 pair-wise contact potential matrices from AAIndex3^63^. The amino acid contact potential difference was obtained by calculating the contact potential difference between the mutant and wild-type residues in the region adjacent to the N/C terminus.

Evolutionary Information: We derived evolutionary features from the Position-Specific Scoring Matrix (PSSM). Specifically, we extracted the wild-type and mutant residue scores at the mutation site and computed their differences to capture mutation-induced changes. The PSSM was generated by performing three iterative searches^65^ against the UniRef90 database^66^ with an E-value threshold of 0.001. To further integrate evolutionary information from neighboring residues, we applied a weighted decay-based local PSSM feature extraction method^67^. The specific procedure is as follows: first, using the sigmoid function:

1 $$h(x)=\frac{1}{1+{e}^{-x}}$$

the values of the original PSSM matrix are normalized to the (0,1) interval to obtain a 20-dimensional feature vector for each residue. Next, three residues to the left and three residues to the right of the mutation site were selected as the central of the mutation site to form a local PSSM matrix containing seven residues (with dimensions of 7 × 20). A Gaussian function was calculated.

2 $$h\left(x\right)=\frac{1}{\sqrt{2\pi }\sigma }\exp \left(-\frac{{(x-\mu )}^{2}}{2{\sigma }^{2}}\right)\left(\mu =0,\sigma =1\right)$$

Calculate the weight vector ${\boldsymbol{w}}={[{w}^{-3},{w}^{-2},{w}^{-1},{w}^{0},{w}^{1},{w}^{2},{w}^{3}]}^{T}$ (where ${w}^{0}$ = 1 and the weights decay exponentially with distance) in the interval [−3,3], and multiply the 20-dimensional features of each residue of the local PSSM matrix by the corresponding weights to obtain the weighted 7 × 20-dimensional local PSSM features. Neighboring residue information: To describe the hydrophobicity and polarity differences in the vicinity of the mutation site, we adopt the local window feature extraction method^6^. Specifically, for each mutation site i, within the window size of 3, 5, 7, 9, 11 (corresponding to the window half-width j = 1,2,3,4,5), the following feature differences are calculated

3 $$\Delta {P}_{{\rm{local}}}={P}_{i}({\rm{mutant}})-\frac{1}{2j+1}\mathop{\sum }\limits_{n=i-j}^{i+j}{P}_{n}({\rm{wild}}-{\rm{type}})$$

where P represents a physicochemical property (hydrophobicity or polarity). We calculated differences for five hydrophobicity and five polarity features, yielding ten features in total. In addition, we classified the amino acids into six categories based on their physicochemical properties: aliphatic (G, A, L, I, V), aromatic (F, Y, W), sulfur-containing (M, C), polar (N, Q, S, T, P), negatively (D, E) charged and positively charged (R, H, K) and calculated the difference between the distributions of each category based on the same window, yielding a total of 30 features.

All of the above features were finally combined into a 537-dimensional feature vector and entered into the fully connected layer for predicting the pathogenicity of membrane protein mutations.

### The architecture of MutDPAL

MutDPAL consists of four main modules: Protein Representation Learning Module, Protein-Disease Representation Fusion Module, Transmembrane Environment Representation Learning Module, and Pathogenicity Classification Module, which are implemented as follows.

### Protein representation learning module

The wild-type and mutant protein features were initially processed by a BiLSTM layer, which capture contextual dependencies bidirectionally (i.e., upstream and downstream of mutation sites), providing a comprehensive understanding of how mutations impact the local protein structure and function. The BiLSTM layer comprised 512 hidden units per direction (i.e., 1024 in total for the bidirectional setting). After processing by the BiLSTM, the features were dimensionality-reduced using a linear layer before being passed to Transformer encoders^68^. The Transformer captured long-range global dependencies within the protein sequence, its multi-head self-attention mechanism allowed the model to focus on different regions of the sequence concurrently, identifying complex interactions across distant amino acids. By combining the local context from BiLSTM and the global dependencies from Transformer, the model comprehensively assesses the mutation’s impact on protein functionality. Formally, the definition of the multi-head self-attention mechanism is as follows:

4 $${\rm{MultiHead}}\left(Q,K,V\right)={\rm{Concat}}\left({hea}{d}_{1},\,\cdots ,{hea}{d}_{h}\right){W}_{O}$$

5 $${hea}{d}_{i}={\rm{Attention}}(Q{W}_{i}^{Q},K{W}_{i}^{K},V{W}_{i}^{V})$$

where the attention mechanism is calculated by:

6 $${\rm{Attention}}\left({Q}_{i},{K}_{i},{V}_{i}\right)={softmax}\left(\frac{{Q}_{i}{K}_{i}^{T}}{\sqrt{{d}_{k}}}\right){V}_{i}$$

Here, $Q$(Query), *K*(Key), and *V*(Value) are vectors derived from the wild-type or mutant protein features, which have been processed through the BiLSTM and linear layers. ${W}_{i}^{Q}$, ${W}_{i}^{K}$ ${\rm{and}}{W}_{i}^{V}$ are the learnable weight matrices for the query, key, and value vectors in the i-th attention head. ${d}_{k}$ denotes the dimension of the query, key and value vectors. It is used for scaling the dot product of ${Q}_{i}$ ${\rm{and}}$ ${K}_{i}^{T}$, ensuring stable gradients during training.

### Protein-disease representation fusion module

Disease features were first encoded through an embedding layer to generate disease-specific representation, referred to as disease embedding. Next, the wild-type and mutant protein embedding were separately merged with the disease embedding using a cross-attention mechanism, capturing the interdependencies between protein sequences and different diseases. These merged features were represented as wild-type and mutant dis_prot embedding. Subsequently, a further fusion using the cross-attention mechanism was performed on wild-type and mutant dis_prot embedding, enabling a deeper understanding of their subtle differences and similarities, represented as attn_dis_prot embedding. This stepwise fusion strategy allowed the model to precisely capture the nuanced changes in features before and after mutations. Additionally, we performed a subtraction operation between the wild-type and mutant dis_prot embedding to further amplify the differences caused by mutations, resulting in the sub_dis_prot embedding. This approach enhanced the clarity and interpretability of the features, which were then used for downstream tasks such as pathogenicity classification and multi-label disease classification. In the cross-attention mechanism, three core inputs are utilized: *Q*, *K*, and *V*. We fused protein-disease representations using the following cross-attention mechanisms:

7 $${CrossAttentio}{n}_{{dp}}={softmax}\left(\frac{{Q}_{d}{K}_{p}^{T}}{\sqrt{{d}_{k}}}\right){V}_{p}$$

8 $${CrossAttentio}{n}_{{wm}}={softmax}\left(\frac{{Q}_{w}{K}_{m}^{T}}{\sqrt{{d}_{k}}}\right){V}_{m}$$

Here, ${Q}_{d}$ and ${Q}_{w}$ represent the disease embedding and wild-type dis_prot embedding, respectively. ${K}_{p}$ and ${V}_{p}$ represent the wild-type/mutant protein embedding, while ${K}_{m}$ and ${V}_{m}$ represent the mutant dis_prot embedding, ${d}_{k}$ is the dimension of the K-vector. This two cross-attentions effectively captures the relationships between mutant protein and disease features for further analysis.

### Transmembrane environment representation learning module

To further enhance the model’s ability to classify mutation pathogenicity in membrane protein scenarios, we introduced a transmembrane environment representation learning module. BioBERT-Based features were processed through both a linear layer and convolutional layers. The linear layer captured global semantic information, while the convolutional layer focused on local features in the transmembrane regions, effectively modeling critical attributes related to transmembrane structures. These two feature sets were then concatenated to form a unified representation, termed transmembrane embedding, offering a more comprehensive characterization of transmembrane protein properties. This multi-level feature integration significantly improves the model’s performance in pathogenicity classification tasks for membrane proteins.

### Pathogenicity classification module

The attn_dis_prot embedding, sub_dis_prot embedding, and transmembrane embedding were integrated into a multimodal feature representation for the final classification task. In the binary pathogenicity classification task, to further amplify the feature differences caused by mutations, we additionally incorporate mutation-sensitivity features. These features are concatenated with the aforementioned features and fed into a multilayer perceptron (MLP)^69^, which output the probability of mutation pathogenicity via a sigmoid function^70^. For the multi-label disease classification task, the MLP output 15 probability scores, each corresponding to a disease category. These scores indicated the likelihood that the mutation was associated with each disease, with higher scores suggesting a stronger association^71^.

By extracting the wild-type and mutant protein features, integrating multimodal protein-disease features, deeply analyzing mutation site’s transmembrane environment features, and designing an interactive pathogenicity classification module, MutDPAL provides a comprehensive understanding of how mutations impact protein function. This approach significantly improves accuracy in both pathogenicity prediction and multi-label disease classification tasks, offering interpretable results and acting as a valuable tool for studying the pathogenicity of membrane protein mutations.

### Model training

Based on the MutDPAL method, we employed the BCELoss as the loss function and utilized the Adam optimizer. The optimal learning rate and batch size were determined for each task: 0.001 and 128 for the pathogenicity prediction task, and 0.0001 and 256 for the multi-label disease classification task, respectively. In addition, to mitigate overfitting during training, we incorporated a Dropout layer with a dropout probability of 0.2. The MutDPAL model was trained for 100 epochs on both tasks, and the best model parameters were saved based on the validation set performance. Detailed information on model configurations and hyperparameters can be found in the Supplementary Table S19.

### Statistics and reproducibility

The implementation was conducted using Python version 3.8.19. All codes are publicly available on GitHub(https://github.com/ljquanlab/MutDPAL), all data and related features are available on Zenodo (10.5281/zenodo.15720529).

## Supplementary information

Supplemental information

Description of Additional Supplementary Files

Supplementary Data 1-3

nr-reporting-summary

## Data Availability

The primary variant data used in this study were obtained from the MutHTP database^32^, which integrates information from sources such as Humsavar, SwissVar^33^, 1000 Genomes^34^, COSMIC^35^, and ClinVar^36^. All processed datasets used to train and evaluate the model are available at Zenodo (10.5281/zenodo.15720529).

## Code availability

The source code for MutDPAL is publicly available at: https://github.com/ljquanlab/MutDPAL. The implementation is based on Python and PyTorch, with details on software versions and dependencies provided in the repository’s README. Hyperparameter settings and training configurations are listed in Supplementary Table S19.
