# Supplementary material for: Prediction of pathogenic mutations in human transmembrane proteins and their associated diseases via utilizing pre-trained Bio-LLMs
Source: Commun Biol. 2025 Jul 15;8:1050. doi: 10.1038/s42003-025-08452-7 (PMC12264167; doi:10.1038/s42003-025-08452-7)
Supplement: Supplementary file 1 — Supplemental information [file 42003_2025_8452_MOESM1_ESM.pdf]

Supplementary Information

Supplementary Tables

Table S1. Comparison on the PathoClassDS

	ACC	AUC	Recall_Bi	Precision_Bi	MCC	F1_Bi
Varipred	0.52	0.69	0.24	0.82	0.21	0.37
TransEFVP	0.59	0.5	**1**	0.59	0	0.75
MutFormer	0.42	0.39	0.43	0.51	-0.17	0.46
AlphaMissense	0.67	0.74	0.58	**0.83**	0.39	0.68
Pred-MutHTP	0.61		0.68	0.7	0.16	0.69
BorodaTM	0.63		0.61	0.86	0.25	0.71
mCSM-membrane	0.7		1	0.7	0	0.82
TMSNP	0.22		0.18	0.54	-0.39	0.27
MutDPAL	**0.74**	**0.81**	0.80	0.78	**0.47**	**0.79**

Note: The best results are indicated in bold, Varipred and MutFormer provide prediction patterns that can be used directly, and we use their prediction patterns to obtain the corresponding results.TransEFVP does not provide a prediction pattern, so we follow their methods and code to re-train and re-test to obtain the prediction results.

Table S2. Comparison of F1_Bi for 14 proteins on the PathoClassDS

	AlphaMissense	MutDPAL
Q14524	0.69	**0.91**
P35498	**0.93**	0.92
P13569	0.84	**0.97**
Q12809	0.78	**0.92**
P51787	0.78	**0.92**
Q92736	0.58	**0.68**
P21817	0.54	**0.81**
O75445	0.44	**0.50**
P35670	0.86	**0.95**
P78363	0.63	**0.89**
Q8WXH0	0	**0.44**
Q9H251	**0.43**	0.25
P08F94	0.3	**0.74**
Q13635	0.26	**0.90**

Note: The best results are indicated in bold.

Table S3. Comparison of mean F1_Bi for all proteins on the PathoClassDS test sets

Methods	AlphaMissense	MutDPAL
Mean_F1_Bi	0.576	**0.772**

Note: The best results are indicated in bold.

Table S4. Comparison of AlphaMissense and MutDPAL in the identical entries

	ACC	AUC	Recall_Bi	Precision_Bi	MCC	F1_Bi
AlphaMissense	0.67	0.74	0.58	**0.83**	0.39	0.68
MutDPAL	**0.74**	**0.81**	**0.79**	0.78	**0.46**	**0.79**

Note: The best results are indicated in bold, AlphaMissense provides a database of predictions for all possible human single amino acid substitutions for subsequent analyses, and we retrieved predictions from it using UniprotID and mutation information, with a total of 3426 fulfilling conditions.

Table S5. Comparison of Pred-MutHTP and MutDPAL in the identical entries

	ACC	AUC	Recall_Bi	Precision_Bi	MCC	F1_Bi
Pred-MutHTP	0.61		0.68	0.7	0.16	0.69
MutDPAL	**0.67**	**0.70**	**0.76**	**0.73**	**0.27**	**0.75**

Note: The best results are indicated in bold, Pred-MutHTP provides a free web server, and we identified variants that were not used in their training, and used UniprotID as well as mutation information, etc., to obtain predictions from this site, with a total of 1813 fulfilling conditions.

Table S6. Comparison of BorodaTM and MutDPAL in the identical entries

	ACC	AUC	Recall_Bi	Precision_Bi	MCC	F1_Bi
BorodaTM	0.63		0.61	0.86	0.25	0.71
MutDPAL	**0.85**	**0.90**	**0.93**	**0.88**	**0.54**	**0.90**

Note: The best results are indicated in bold, BorodaTM provided all the predictions for subsequent analyses, and we retrieved predictions from this method using UniprotID as well as mutation information, with a total of 272 fulfilling conditions.

Table S7. Comparison of mCSM-membrane and MutDPAL in the identical entries

	ACC	AUC	Recall_Bi	Precision_Bi	MCC	F1_Bi
mCSM-membrane	0.7		**1**	0.7	0	0.82
MutDPAL	**0.78**	**0.82**	0.86	**0.83**	**0.47**	**0.84**

Note: The best results are indicated in bold, mCSM-membrane provides a free web server, and we used UniprotID, mutation information, and pdbID to get predictions from this site, with a total of 1688 fulfilling conditions.

Table S8. Comparison of TMSNP and MutDPAL in the identical entries

	ACC	AUC	Recall_Bi	Precision_Bi	MCC	F1_Bi
TMSNP	0.22		0.18	0.54	-0.39	0.27
MutDPAL	**0.81**	**0.77**	**0.89**	**0.88**	**0.42**	**0.88**

Note: The best results are indicated in bold, TMSNP provides a free web server to predict only variants whose mutations are in the transmembrane region, and we used UniprotID as well as mutation information, etc. to get prediction results from this site, with a total of 227 fulfilling conditions.

Table S9. Comparison within transmembrane regions on the PathoClassDS

	ACC	AUC	Recall_Bi	Precision_Bi	MCC	F1_Bi
Varipred	0.53	0.73	0.38	0.83	0.22	0.52
TransEFVP	0.67	0.52	**1**	0.67	0	0.80
MutFormer	0.34	0.30	0.34	0.52	-0.29	0.41
AlphaMissense	0.78	0.82	0.81	**0.86**	0.52	0.84
Pred-MutHTP	0.68		0.75	0.80	0.23	0.77
MutDPAL	**0.80**	**0.85**	0.87	0.84	**0.53**	**0.85**

Note: The best results are indicated in bold, Varipred, TransEFVP, MutFormer, MutDPAL with 679 samples, Pred-MutHTP with 280 samples fulfilling the condition, Alphamissense with 631 samples fulfilling the condition.

Table S10. Comparison within cytoplasmic regions on the PathoClassDS

	ACC	AUC	Recall_Bi	Precision_Bi	MCC	F1_Bi
Varipred	0.48	0.64	0.20	**0.80**	0.17	0.32
TransEFVP	0.61	0.50	**1**	0.61	0	**0.76**
MutFormer	0.47	0.46	0.45	0.58	-0.06	0.50
AlphaMissense	0.61	0.67	0.49	0.80	0.30	0.61
Pred-MutHTP	0.55		0.63	0.63	0.05	0.63
MutDPAL	**0.70**	**0.75**	0.76	0.75	**0.37**	0.75

Note: The best results are indicated in bold, Varipred, TransEFVP, MutFormer, MutDPAL with 1506 samples, Pred-MutHTP with 820 samples fulfilling the condition, Alphamissense with 1382 samples fulfilling the condition.

Table S11. Comparison within extracellular regions on the PathoClassDS

	ACC	AUC	Recall_Bi	Precision_Bi	MCC	F1_Bi
Varipred	0.54	0.70	0.21	0.82	0.22	0.34
TransEFVP	0.55	0.49	**1**	0.55	0	0.71
MutFormer	0.40	0.37	0.45	0.46	-0.20	0.45
AlphaMissense	0.68	0.75	0.55	**0.83**	0.42	0.66
Pred-MutHTP	0.65		0.70	0.73	0.25	0.72
MutDPAL	**0.76**	**0.83**	0.80	0.77	**0.52**	**0.79**

Note: The best results are indicated in bold, Varipred, TransEFVP, MutFormer, MutDPAL with 1622 samples, Pred-MutHTP with 713 samples fulfilling the condition, Alphamissense with 1413 samples fulfilling the condition.

Table S12. Comparison of AUC values for 15 diseases

	DT	ET	ETs	KNN	MLP	RF	Varipred	TranEFVP	MutFormer	MutDPAL
NSD	0.7714	0.7491	0.959	0.8593	0.9579	0.9611	0.8258	0.5	0.8842	**0.9703**
DSD	0.7328	0.7199	0.9258	0.8079	0.9075	0.8966	0.8341	0.5	0.9565	**0.9728**
OCD	0.694	0.6946	0.9266	0.8078	0.921	0.9242	0.7979	0.5	0.9144	**0.9445**
CDM	0.8638	0.8367	0.9763	0.9121	0.9699	0.9771	0.9311	0.5	0.9242	**0.98**
RSD	0.7074	0.5822	0.9989	0.8321	**0.9994**	0.9982	0.8655	0.5	0.9323	0.9993
CD	0.8035	0.7688	0.9708	0.9059	0.9682	0.9741	0.8789	0.5	0.9523	**0.9786**
RD	0.6241	0.6246	0.8628	0.7465	0.9391	0.863	0.7853	0.5	**0.9997**	0.8351
ISD	0.8473	0.8006	0.9773	0.86	**0.9839**	0.9773	0.9444	0.5	0.9526	0.9702
EMD	0.741	0.7385	0.9794	0.8478	0.9661	0.9694	0.8765	0.5	0.9567	**0.9849**
MD	0.7999	0.7772	0.9886	0.8699	0.9801	0.9804	0.8398	0.5	0.9366	**0.9941**
USD	0.804	0.7523	0.954	0.8602	0.9603	0.957	0.9129	0.5	0.9683	**0.9860**
SD	0.7739	0.7583	0.9588	0.8339	0.9585	0.9603	0.865	0.5	0.9426	**0.9735**
CN	0.5512	0.5566	0.6644	0.6209	0.6636	0.6725	0.6996	0.5	**0.775**	0.6833
NP	0.5738	0.5328	0.719	0.6468	**0.7818**	0.7265	0.7482	0.5	0.7797	0.7602
UN	0.59	0.5598	0.6836	0.6154	**0.8846**	0.751	0.7522	0.5	0.8816	0.8762

Note: The best results are indicated in bold.

Table S13. Comparison of AUPR values for 15 diseases

	DT	ET	ETs	KNN	MLP	RF	Varipred	TranEFVP	MutFormer	MutDPAL
NSD	0.5227	0.4916	0.869	0.6782	0.8879	0.8832	0.644	0.1447	0.599	**0.9091**
DSD	0.2456	0.2329	0.6477	0.3929	0.6338	0.6523	0.5009	0.0111	0.3004	**0.7784**
OCD	0.2042	0.1981	0.588	0.3083	0.6073	0.5894	0.351	0.0548	0.4341	**0.7123**
CDM	0.6613	0.6087	0.9348	0.8004	0.9378	0.9398	0.8437	0.1307	0.7372	**0.9494**
RSD	0.2346	0.052	0.7533	0.5163	**0.9052**	0.7364	0.1889	0.0008	0.2982	0.8457
CD	0.5177	0.4409	0.8728	0.6916	0.8926	0.8776	0.6388	0.0836	0.699	**0.8956**
RD	0.0513	0.0847	0.5838	0.2726	0.6268	0.3192	0.6047	0.0013	**0.7556**	0.6882
ISD	0.4681	0.3759	**0.8316**	0.549	0.7983	0.8273	0.5542	0.0137	0.4413	0.8170
EMD	0.3086	0.2778	0.7663	0.4796	0.7132	0.7559	0.4065	0.0295	0.3747	**0.8315**
MD	0.4157	0.3465	0.8991	0.5099	0.8895	0.8799	0.4464	0.0559	0.5984	**0.9179**
USD	0.383	0.2773	0.7638	0.5443	0.7429	0.7611	0.5678	0.0269	0.5944	**0.8349**
SD	0.349	0.34	0.8048	0.5407	0.7963	0.8153	0.5021	0.0271	0.4422	**0.8498**
CN	0.1482	0.151	0.2749	0.2374	0.2896	0.2991	0.2726	0.083	0.2421	**0.3572**
NP	0.0699	0.0521	0.1362	0.1013	**0.1862**	0.147	0.1571	0.0271	0.1237	0.1711
UN	0.0338	0.024	0.1273	0.0982	0.1117	0.1299	0.1069	0.0063	0.3323	**0.3582**

Note: The best results are indicated in bold.

Table S14. Comparison in terms of multi-label metrics with MutDPAL_netural

Model	Precision_ML(↑)	Recall_ML(↑)	F1_ML(↑)	Hamming Loss(↓)
MutDPAL_netural	0.63	0.62	0.62	0.04
MutDPAL	**0.78**	**0.76**	**0.76**	**0.03**

Note: The best results are indicated in bold, MutDPAL_netural indicates the inclusion of a neutral sample as a control.

Table S15. Comparison of single-label cases

Model	Precision_ML(↑)	Recall_ML(↑)	F1_ML(↑)	Hamming Loss(↓)
DT	0.54	0.65	0.57	0.06
ET	0.49	0.61	0.53	0.07
ETs	0.53	0.61	0.56	**0.03**
KNN	0.50	0.55	0.52	0.04
MLP	0.67	0.73	0.69	**0.03**
RF	0.51	0.58	0.54	**0.03**
Varipred	0.41	0.41	0.41	0.05
TransEFVP	0	0	0	0.07
MutFormer	0.41	0.41	0.41	0.05
MutDPAL	**0.77**	**0.80**	**0.78**	**0.03**

Note: The best results are indicated in bold.

Table S16. Comparison of multi-label cases

Model	Precision_ML(↑)	Recall_ML(↑)	F1_ML(↑)	Hamming Loss(↓)
DT	0.63	0.41	0.48	0.11
ET	0.61	0.41	0.47	0.11
ETs	0.65	0.40	0.49	0.09
KNN	0.56	0.33	0.41	0.12
MLP	0.78	0.46	0.56	0.09
RF	0.63	0.38	0.47	0.10
Varipred	0.43	0.22	0.29	0.12
TransEFVP	0	0	0	0.14
MutFormer	0.37	0.20	0.26	0.12
MutDPAL	**0.86**	**0.51**	**0.62**	**0.08**

Note: The best results are indicated in bold.

Table S17. Some data examples in the PathoClassDS

UniprotID	Mutation	Ref	pos	Mut	Disease	Dis_class
Q14524	A1113V	A	1112	V	Brugada syndrome 1	cardiovascular diseases
Q14643	V1562M	V	1561	M	Spinocerebellar ataxia 29	nervous system diseases
Q14654	R50Q	R	49	Q	Neonatal diabetes mellitus	endocrine and metabolic diseases
P03915	D503G	D	502	G	Netural	-
P04626	G815R	G	814	R	Neutral	-

Table S18. The number of 15 disease classes in the DiseaseClassDS

	NSD	DSD	OCD	CDM	RSD	CD	RD	ISD	EMD	MD	USD	SD	CN	NP	UN
All	5418	365	1857	4917	67	3308	34	565	983	2183	889	1063	3266	1025	153
Train	4318	287	1512	3932	46	2622	29	450	780	1792	715	834	2633	829	122
Valid	533	40	189	486	9	329	1	61	99	207	83	110	337	92	15
Test	567	38	156	499	12	357	4	54	104	184	91	119	296	104	16

Table S19.List of hyperparameters.

Name	Value1	Value2
Dimension of protein features	1493	1493
Number of Bi-LSTM layers	1	1
Hidden size	1024	1024
Number of Transformer encoder layers	4	4
Learning rate	0.01	0.0001
Batch size	128	512
Dropout	0.2	0.2

Note: Value1 represents the pathogenicity classification task and value2 represents the multi-label disease classification task.

Supplementary Texts

Text S1. Evaluation metrics for pathogenicity classification models.

$$ACC = \frac{TP + TN}{TP + TN + FP + FN}$$

$$Recall\_Bi = \frac{TP}{TP+FN} \qquad Precision\_Bi = \frac{TP}{TP+FP}$$

$$F1\_Bi = \frac{2 * Precision * recall}{Precision + recall}$$

$$MCC = \frac{TP \cdot TN \cdot FP \cdot FN}{\sqrt{(TP + FN)(TP + FP)(TN + FP)(TN + FN)}}$$

$$Specificity = \frac{TN}{TN+FP}$$

TP represents true positive samples, TN represents true negative samples, FP represents false positive samples, and FN represents false negative samples. The MCC provides a comprehensive evaluation by considering all four classification outcomes, while the F1-score combines precision and recall, making it particularly useful for handling imbalanced data.

Text S2. Evaluation metrics for multi-label disease classification models.

$$F1\_ML = \frac{1}{n}\sum_{i=1}^{n} \frac{2|Y_i \cap Z_i|}{|Y_i| + |Z_i|}$$

$$Recall\_ML = \frac{1}{n}\sum_{i=1}^{n} \frac{|Y_i \cap Z_i|}{|Y_i|}$$

$$Precision\_ML = \frac{1}{n}\sum_{i=1}^{n} \frac{|Y_i \cap Z_i|}{|Z_i|}$$

$$HammingLoss = \frac{1}{n}\sum_{i=1}^{n} \frac{|Y_i \oplus Z_i|}{n_{class}}$$

Y_i denotes the true labels for sample i in the dataset, Z_i indicates the predicted labels for sample i, $|Y_i|$ represents the count of true labels equal to 1 for sample i, and $|Z_i|$ signifies the count of predicted labels equal to 1 for sample i. Here, n represents the total number of samples in the test set, and n_{class} indicates the number of labels for each sample.

Text S3. AAindex1 feature filtering process

1. Data Source:

   We utilized the AAIndex1 database, which contains 566 physicochemical properties of amino acids[1]. Each property entry includes a correlation field (Field C) listing identifiers of other properties with high correlation (precomputed by the database).

2. Correlation Filtering Strategy:

   (1) Initialization: Two sets were maintained

   - Set A: Final selected properties (initially empty).

   - Set B: A "blocklist" of property identifiers to exclude due to correlation with previously selected properties (initially empty).

   (2) Iterative Process:

   - For each property in the database, we checked if its identifier (Field H) was already in Set B.

   - If present, the property was skipped to avoid redundancy.

   - If absent, the property was added to Set A, and all correlated property identifiers from its Field C were added to Set B to block subsequent selection of highly correlated properties.

   (3) Result: Only the first-occurring representative of a correlated group was retained, ensuring minimal redundancy.

3. Outcome:

   This method reduced the original 566 properties to 213 non-redundant properties, effectively eliminating highly correlated features while preserving representative physicochemical attributes.

Supplementary Figures

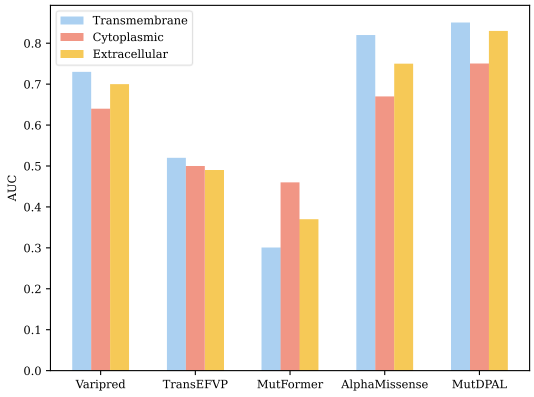

Figure S1. AUC score by model and region.

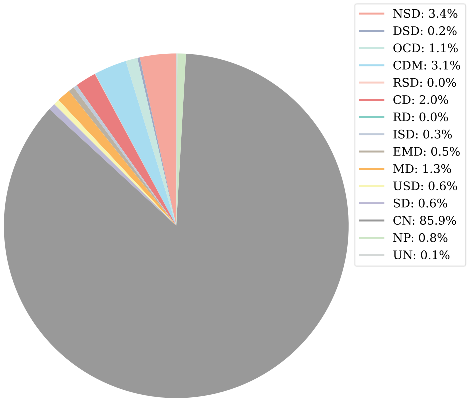

Figure S2. Percentage of disease-causing mutations in MutHTP by disease category.

Reference

1. Kawashima S, Ogata H, Kanehisa M. AAindex: amino acid index database. *Nucleic acids research* **27**, 368-369 (1999).
