# Supplementary material for: Prediction of pathogenic mutations in human transmembrane proteins and their associated diseases via utilizing pre-trained Bio-LLMs
Source: Commun Biol. 2025 Jul 15;8:1050. doi: 10.1038/s42003-025-08452-7 (PMC12264167; doi:10.1038/s42003-025-08452-7)
Supplement: Supplementary file 2 — Description of Additional Supplementary Files [file 42003_2025_8452_MOESM2_ESM.pdf]

Description of Additional Supplementary Files

File name: Supplementary Data 1

Description: The source data behind the Figure 2、3、5、7 in the paper.

├── Sheet1 ： Figure2 plot data

├── Sheet2 ： Figure3 plot data

├── Sheet3 ： Figure5 plot data

├── Sheet4 ： Figure7 plot data

File name: Supplementary Data 2

Description: The source data behind Figure 4 in the paper

├── Sheet1 ： Figure 4(B) plot data

├── Sheet2 ： Figure 4(C) plot data

File name: Supplementary Data 3

Description: The source data behind Figure 6 in the paper.

├── Sheet1 ： Figure 6(A-B) plot data

├── Sheet2 ： Figure 6(C) plot data

├── Sheet3 ： Figure 6(D) plot data

├── Sheet4 ： Figure 6(E) plot data

├── Sheet5 ： Figure 6(F) plot data
